# Case Report: Extramedullary Acute Promyelocytic Leukemia: An Unusual Case and Mini-Review of the Literature

**DOI:** 10.3389/fonc.2022.886436

**Published:** 2022-05-25

**Authors:** Dennis Christoph Harrer, Florian Lüke, Ingo Einspieler, Karin Menhart, Dirk Hellwig, Kirsten Utpatel, Wolfgang Herr, Albrecht Reichle, Daniel Heudobler

**Affiliations:** ^1^ Department of Internal Medicine III, Hematology and Oncology, University Hospital Regensburg, Regensburg, Germany; ^2^ Institute of Radiology, University Hospital Regensburg, Regensburg, Germany; ^3^ Department of Nuclear Medicine, University Hospital of Regensburg, Regensburg, Germany; ^4^ Institute of Pathology, University of Regensburg, Regensburg, Germany; ^5^ Bavarian Cancer Research Center (BZKF), Regensburg, Germany

**Keywords:** acute promyelocytic leukemia, chloroma, myeloid sarcoma, PML-RARA rearrangement, normal blood counts

## Abstract

**Background:**

Acute promyelocytic leukemia (APL) constitutes a serious hematological emergency necessitating rapid diagnosis and therapy to prevent lethal bleedings resulting from APL-induced thrombocytopenia and coagulopathy. Atypical manifestations of APL, such as extramedullary disease at first presentation, pose diagnostic challenges and delay the onset of appropriate therapy. Nevertheless, extramedullary manifestations of APL are mostly accompanied by blood count alterations pointing to an underlying hematological disease. In this report, we present the first case of APL bearing close resemblance to a metastasized laryngeal carcinoma with normal blood counts and absent coagulopathy.

**Case Presentation:**

A 67-year-old man with a previous history of smoking was admitted to our hospital with progressive hoarseness of voice, odynophagia, dysphagia and exertional dyspnea. Laryngoscopy revealed a fixed right hemi larynx with an immobile right vocal fold. Imaging of the neck *via* magnetic-resonance imaging (MRI) and positron emission tomography–computed tomography (PET/CT) with F-18-fluordeoxyglucose (FDG) showed a large hypermetabolic tumor in the right piriform sinus and tracer uptake in adjacent lymph nodes, highly suspicious of metastasized laryngeal carcinoma. Surprisingly the histological examination revealed an extramedullary manifestation of acute promyelocytic leukemia. Remarkably, blood counts and coagulation parameters were normal. Moreover, no clinical signs of hemorrhage were found. *PML-RARA* fusion was detected in both laryngeal mass and bone marrow. After diagnosis of APL, ATRA-based chemotherapy was initiated resulting in complete remission of all APL manifestations.

**Conclusions:**

This is the first case report of APL initially presenting as laryngeal chloroma. Additionally, we performed a comprehensive literature review of previously published extramedullary APL manifestations. In aggregate, a normal blood count at first presentation constitutes an extremely rare finding in patients initially presenting with extramedullary APL manifestations.

## Background

Formerly classified as AML M3 according to the French-American-British (FAB) classification (1), acute promyelocytic leukemia (APL) is currently listed as AML with certain genetic abnormalities by the world health organization (WHO) (2). The *PML-RARA* fusion gene originating from a reciprocal translocation connecting the long arms of chromosomes 15 and 17 [t(15;17)(q24.1;q21.1)] constitutes the signature genetic alteration of APL (2). In rare cases, other aberrations, such as t(11;17), t(5;17) and the presence of *PML-RARA* without an underlying translocation were reported (3). Mechanistically, these chromosomal alterations disrupt the *RARA* gene located on chromosome 17 resulting in a differentiation arrest at the promyelocytic stage (3).

The initial diagnosis of APL, which accounts for 5-10% of all newly diagnosed AML cases, poses a critical hematological emergency (4). The high early mortality rate of APL patients frequently arises from APL-induced thrombocytopenia and coagulopathy causing lethal cranial or pulmonary hemorrhage (4). After surviving the initial phase, risk-stratified treatment regimens incorporating the vitamin A-derivative all-trans retinoic acid (ATRA), the inorganic compound arsenic trioxide (ATO), anthracyclines and cytarabine have dramatically improved the overall survival of APL patients (4). Hence, a succinct diagnostic process and the rapid commencement of induction chemotherapy with an ATRA-based combination are crucial factors in reducing early mortality and improving the overall-survival of APL patients.

Atypical manifestations of APL compound swift diagnosis and delay the initiation of induction chemotherapy. Recently, a rare case of extensive extramedullary APL lesions with infiltration of the clivus, mandible and maxilla was published. Nevertheless, pancytopenia, coagulation alterations and characteristic bone marrow findings enabled early diagnosis of APL (5).

We present another atypical case of APL bearing close resemblance to a locally metastasized laryngeal carcinoma. In this case, however, normal blood counts, normal coagulation parameters and a discontinuous bone marrow involvement causing false negative bone marrow aspiration results additionally complicated APL-diagnosis. The aim of this case report is to highlight unprecedented obstacles to swift APL diagnosis.

## Case Presentation

**Figure 1:**
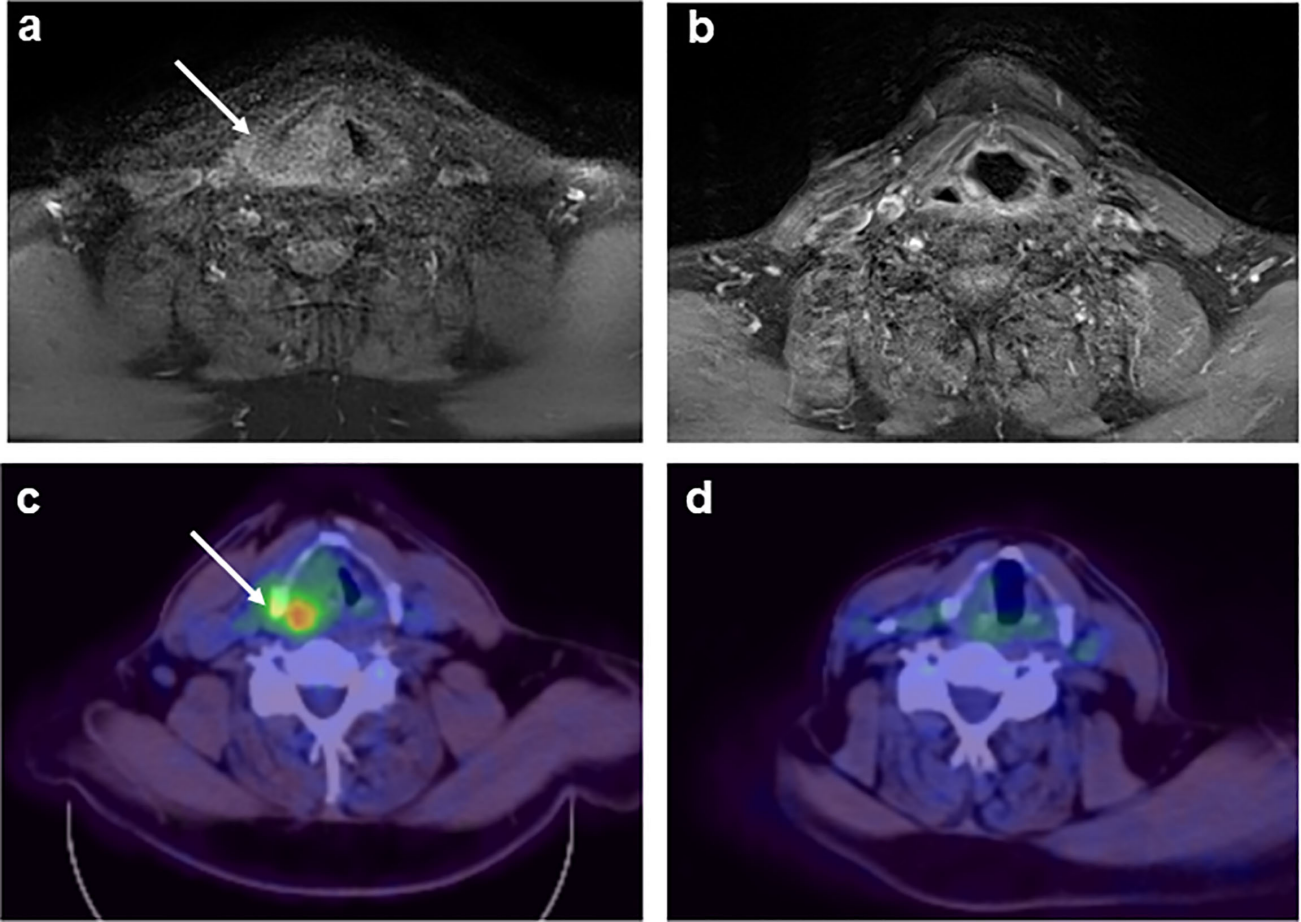
MRI **(A, B)** and 18-FDG-PET **(C, D)** of the neck displaying a laryngeal tumor mass at initial diagnosis [**(A, C)**; arrow] and complete remission of the tumor formation after induction + consolidation I **(B, D)**.

**Figure 2:**
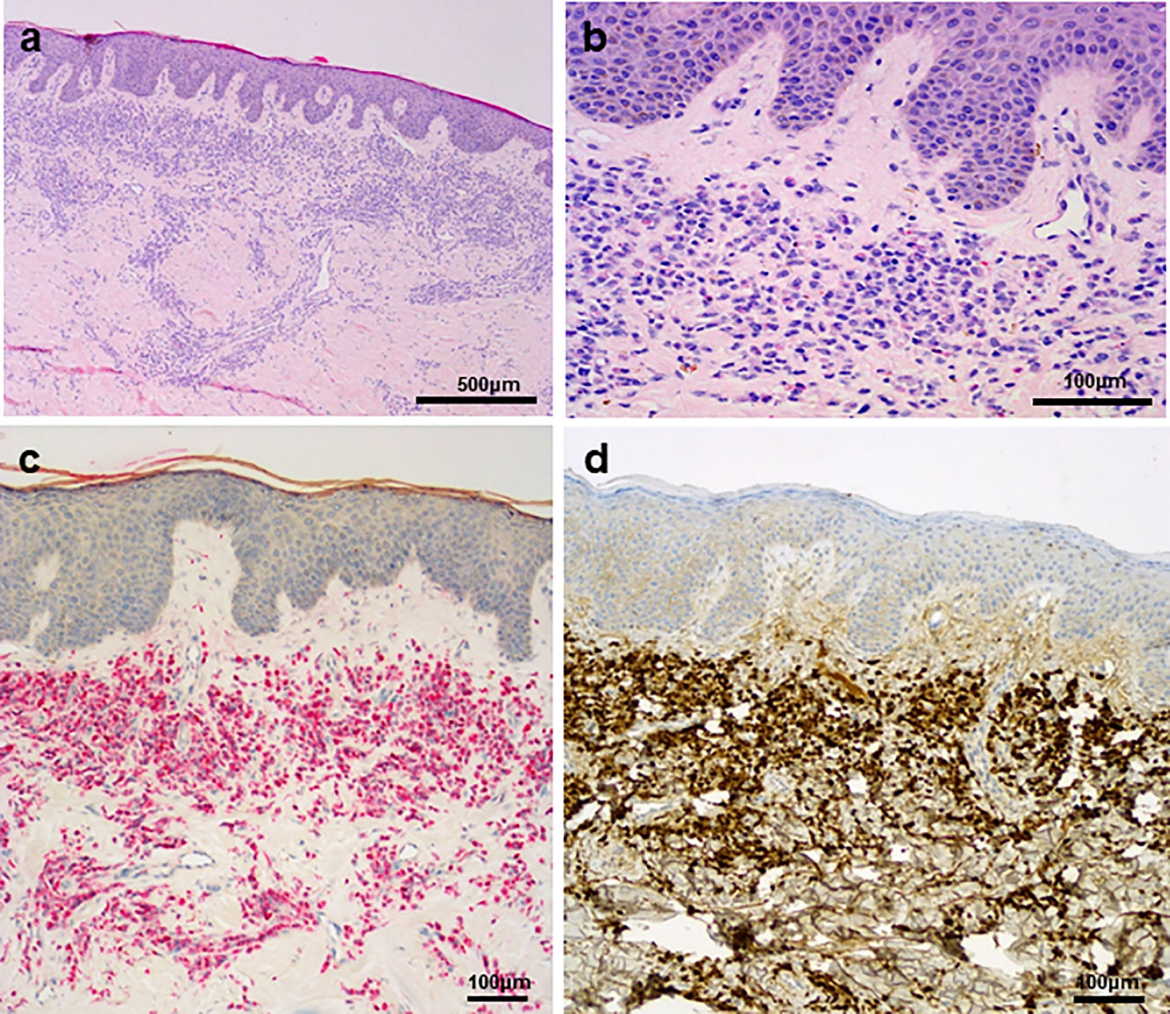
**(A)** Skin biopsy with lichenoid leukaemic infiltration in the upper dermis. **(B)** Grooved nuclei and granulated eosinophilic cytoplasm are visible in the blasts. **(C, D)** There is a strong chloroacetate esterase reaction **(C)** in the infiltrate, as well as MPO reaction **(D)**.

**Figure 3:**
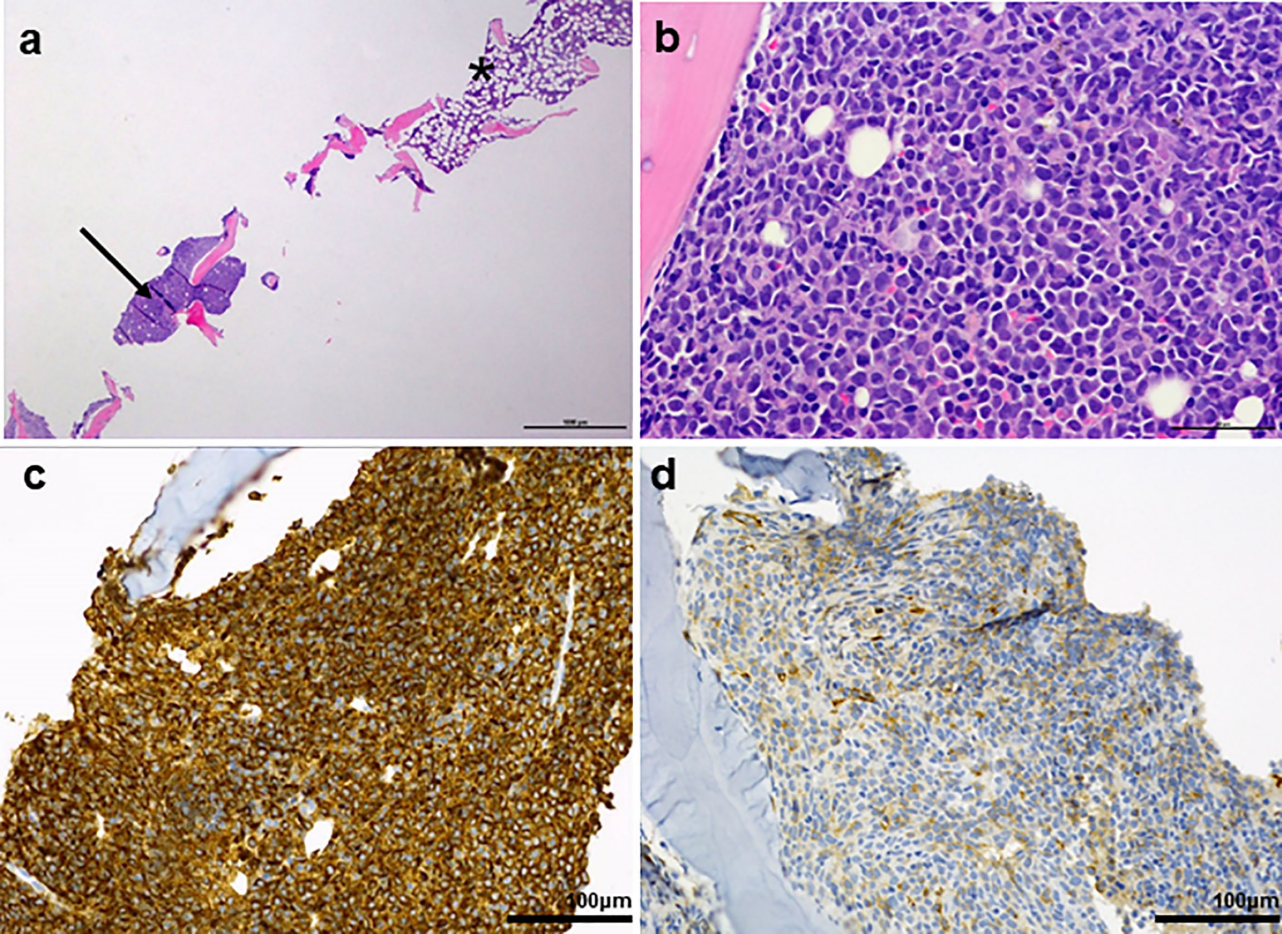
**(A)** Overview of a bone marrow biopsy with discontinuous infiltration by acute promyelocytic leukemia (arrow: APL; *: areas with normal bone marrow. **(B)** Abnormal promyelocyctes show variable nuclear size and shape. Typical kidney shaped nuclei are visible. **(C)** Strong MPO reaction in all leukaemic promyelocyctes. **(D)** Partial and weak CD34 expression.

A 67-year-old man with a 2-month history of progressive hoarseness of voice, odynophagia, dysphagia, and exertional dyspnea presented to our hospital. In addition, he complained about a new growing skin lesion on his back. The patient had a previous history of smoking, but he reported ongoing abstinence from nicotine for 17 years. In the clinical examination, several subcutaneous nodules located on the right chest, on the head and on the back were found. Laryngoscopy revealed a fixed right hemilarynx with an immobile right vocal fold and a normal left vocal fold. Magnetic-resonance imaging (MRI) of the neck showed a large mass protruding into the right hemilarynx (**Figure 1A**, arrow). On suspicion of laryngeal carcinoma with cutaneous metastases, a positron emission tomography–computed tomography (PET/CT) scan with F-18-fluordeoxyglucose (FDG) was performed. The right piriform sinus and adjacent cervical lymph nodes showed significant tracer uptake (**Figure 1C**, arrow). Subsequently, a pharyngeal pan-endoscopy revealed the presence of a large tumor mass originating from the right piriform sinus, which was highly suggestive of carcinoma. Histopathological examination, on the other hand, disclosed infiltrates of atypical blastoid cells growing incohesively beneath an intact epithelial layer. Within those blastoid cells, immunohistochemistry demonstrated high expression of CD45 and myeloperoxidase (MPO). The pan-cytokeratin antibody only reacted with squamous epithelial cells, indicating that squamous cell carcinoma was not present. Similarly, skin biopsies removed from the back confirmed infiltrates of blastoid cells that expressed CD45, chloroacetate esterase, and MPO (**Figures 2A–D**). Collectively, the lesion suspicious for larynx carcinoma and the skin lesions were identified as leukemic infiltrates, termed chloromas, of an acute myeloid leukemia. For further diagnostic and therapeutic steps, the patient was referred to the department of hematology. At admission, blood counts, coagulation parameters and LDH were within normal limits [white blood cell count: 6.50/nl (normal range: 4.23-9.1), hemoglobin level: 16.3 g/dl (normal range: 13.7-17.5), platelet count: 156/nl (normal range: 163-337)]. No blasts were found in the peripheral blood. The ensuing bone marrow examination yielded equivocal results: While neither microscopy nor flow cytometry immunophenotyping could confirm the presence of leukemia in the bone marrow aspirate, histopathological examination of the bone marrow biopsy revealed a significant infiltration with 50% myeloid blasts. Remarkably, the bone marrow morphology exhibited a strict dichotomy between areas exclusively covered with blastoid cells (**Figures 3A, B**) expressing MPO (**Figure 3C**) and areas with normal hematopoiesis (**Figure 3A**) consisting of chloroacetate esterase expressing granulocytes, CD61-positive megakaryocytes and confluent clusters of regular erythropoesis. Furthermore, 20% of the blastoid cells were positive for CD34 (**Figure 3D**), but no CD45, CD61, CD68 or chloracetate esterase expression could be detected. The blastiod cells were embedded in CD68-positive reticulum cells, and reticular fibres. Using nested PCR weak signals of *PML-RARA* fusion transcripts were found. The cytogenetical analysis yielded a normal male karyotype with no apparent alterations. Notably, no translocation t(15;17) could be detected *via* fluorescence *in situ* hybridization (FISH). Due to the low signal intensity of *PML-RARA*, additional molecular genetic examinations of peripheral blood and skin chloromas were performed. The *PML-RARA* fusion was detected by nested PCR in both blood and skin samples, confirming the diagnosis of APL with cryptic *PML-RARA* rearrangement.

## Treatment and Outcome

After initial diagnosis of AML, the patient was subjected to induction chemotherapy consisting of cytarabine (200 mg absolute, day 1-7) and daunorubicin (120 mg absolute, d3-5). Having corroborated the diagnosis of APL based on the evidence of *PML-RARA* rearrangement in several independent samples, the treatment regimen was switched to ATRA. Two weeks after the initiation of ATRA, *PML-RARA* was no longer detectable in the bone marrow. *PML-RARA* intensity in the skin chloromas exhibited a decrease by 95%. After regeneration of blood counts the patient underwent the first cycle of consolidation chemotherapy with high-dose cytarabine, ATRA and idarubicin according to the AIDA-2000 protocol for high-risk APL (6). At the beginning of the second consolidation cycle with ATRA, mitoxantrone and etoposide the laryngeal mass and the skin chloromas had almost completely vanished. Before the start of the third cycle of consolidation with ATRA, idarubicin, 6-thioguanine and cytarabine, PCR analysis of skin chloromas did not show any residual *PML-RARA* signals. After three rounds of consolidation therapy, a complete regression of APL manifestations was observed with negative *PML-RARA* PCR results in bone marrow and skin biopsies. Besides, MRI and FDG-PET/CT imaging revealed complete remission of the laryngeal chloroma (**Figures 1B, D**). The patient underwent maintenance therapy consisting of methotrexate, 6-thioguanine and ATRA for two years with regular remission controls by bone marrow examination every three months. The last remission control conducted two years after termination of maintenance therapy indicated ongoing complete remission with complete absence of PML-RARA transcripts.

## Discussion and Conclusion

Acute promyelocytic leukemia is a unique subgroup of AML displaying a variety of distinct features regarding diagnosis and treatment. Patients with APL usually present with abnormal blood counts, such as leukocytosis, leukocytopenia, thrombocytopenia and anemia (7). Thrombocytopenia coupled with hypofibrinogenemia and disseminated intravascular coagulation poses a serious jeopardy for APL patients necessitating early intervention to prevent lethal cerebral or pulmonary bleedings (7). Approximately 25% of APL patients evince a thrombocyte count below 40/nl (8). The hemorrhage rate at diagnosis can be estimated at 75% with lethal outcomes in less than 10% of APL patients (8). Mechanistically, thrombocytopenia is, among other factors, rationalized by bone marrow invasion of the leukemic clone. The dysfunctionality of the plasmatic coagulation system is largely ascribed to hyperfibrinolysis induced by APL cells (8). Additionally, myeloblasts are frequently found in the peripheral blood at initial diagnosis. Most of the time, the presence of APL can be confirmed by microscopy of bone marrow aspirate showing expansion of myeloblasts and atypic promyelocytes. Whereas the presence of *PML-RARA* is a vital precondition for the diagnosis APL, the signature translocation t(15;17) is found in more than 90% of all APL cases, but it does not constitute an essential diagnostic criterion (3). Chloromas are rather rare at first presentation but occur more frequently in patients with relapsing disease (9–13). To date, only few reports on primary extramedullary manifestation of APL exist (5, 14–29), e.g. the clinical courses of a 29-year-old male presenting with hematochezia resulting from a chloroma in the colon (14), and a 38-year-old female patient complaining about gingival bleeding, abnormal uterine bleeding and headache emanating from an extensive intracranial chloroma. Of note, in both cases pancytopenia prompted bone marrow examination yielding extensive expansion of blasts and proof of *PML-RARA* translocation confirming the presence of APL. In this case report, however, the patient initially presented with normal blood counts and coagulation parameters, which to our knowledge has never been published in adults (>21 years) before (**Table 1**). Hence, against the backdrop of a previous history of smoking the cause for the reported symptoms was rather assumed to be in the pharyngeal/laryngeal area, which was further underpinned by radiological findings implying laryngeal carcinoma with nodular and skin metastases. Intricately, having identified the skin alterations as chloromas, the ensuing cytological and flow cytometric analysis of bone marrow aspirate returned false negative results. The reason for this was evident from the histopathological examination showing a strikingly heterogenous distribution of malignant and benign cells to well demarcated areas. Finally, a cryptic *PML-RARA* rearrangement without an underlying t(15;17) did further delay the diagnosis of APL, as the weak intensity of the *PML-RARA* signal required a second confirmatory PCR. Usually, a protracted onset of chemotherapy diminishes the survival of patients with APL by raising the chances for serious hemorrhage. Fortunately, in this case, the initial diagnosis of APL was complicated by absent hematological abnormalities reducing the jeopardy for acute incidents of serious bleeding. Due to the pronounced extramedullary leukemia formation, the patient was assigned to the high-risk group of APL patients, albeit all blood counts were within normal range. This procedure is in line with a previous case report about a patient exhibiting extensive intracranial infiltration of APL, who was subjected to intensified therapy despite low risk disease according to Sanz risk score calculated from peripheral leukocyte and thrombocyte count (5). Presenting with a laryngeal mass and subcutaneous nodules located on the head one might also suspect potential central nervous system (CNS) involvement in our patient. In this context, intracranial manifestion of APL at diagnosis is extremely rare and has only been reported in few cases (5, 30, 31). Furthermore, the European LeukemiaNet (ELN) guideline on the diagnosis and management of AML in adults does not recommend intrathecal prophylaxis in patients without CNS symptoms (32, 33). Accordingly, cerebrospinal fluid (CSF) analysis and intrathecal prophylaxis were not performed in the presented case. Yet, high-dose cytarabine was given for consolidation according to the AIDA-2000 protocol for high-risk APL which is effective in CNS leukemia due to its high CSF penetration (34).

**Table 1:** Comparison of case reports about primary extramedullary manifestations in APL reported in the literature.

Author	Age (y), gender	Main symptoms	Chloroma location	Blood counts^a^	Coagulopathy^a^	Bone marrow^a^
present study	67, M	hoarseness of voice, dysphagia	pririform sinus	**normal**	**absent**	PML-RARA
Sticco K et al. (5)	39, F	bleeding, headache	intracranial	pancytopenia	Hypofibrinogenemia elevated D-dimers	t(15;17)
Damodar S et al. (14)	29, M	fever, hematochezia	rectal	pancytopenia	hypofibrinogenemia	t(15;17)
Benjazia E et al. (15)	17, F	hematochezia, abdominal pain	rectal	anemia, thrombocytopenia	n.s.^b^	t(15;17)
Ko MW et al. (16)	54, F	fatigue, vision impairments	optic nerve	leukopenia	n.s.	t(15;17)
Fukushima S et al. (17)	39, F	cerebellar ataxia	cerebellum	leukocytosis, thrombocytopenia	hypofibrinogenemia	PML-RARA
Kyaw TZ et al. (18)	26, M	back pain, paraparesis	spinal	pancytopenia	absent	PML-RARA
Doucet ME et al. (19)	23, F	paraparesis	spinal	thrombocytopenia, anemia	n.s.	t(15;17)
Winters C et a (20).	59, F	back pain	vertebral (L3-S1)	leukopenia	absent	t(15;17)
Thomas X et al. (21)	19, M	sternal pain, fever	sternum	**normal**	**absent**	**not affected**
Nair M et al. (22)	7, M	hip pain, fever	hip, multiple osteolytic lesions	anemia, thrombocytopenia	n.s.	t(15;17)
Worch J et al. (23)	16, F	shoulder pain	shoulder, multiple osteolytic lesions	anemia	n.s.	PML-RARA
Savranlar A et al. (24)	18, M	fever, paraparesis	spinal	anemia, thrombocytopenia	absent	t(15;17)
Stankova J et al. (25)	14, M	paraparesis	spinal	**normal**	**absent**	myeloblasts with Auer rods, no PML-RARA
Shah NN et al. (27)	56, M	back pain	spinal	leukopenia	**absent**	t(15;17)
Collinge E et al. (26)	49, W	fever, bulky skin lesion	skin	hyperleukocytosis, thrombocytopenia	present	t(15;17)

a at first diagnosis;

b n.s., not stated. normal = within reference range, absent = no coagulopathy occurred, not affected = not involved.

In aggregate, we report the first case of APL initially manifesting with a laryngeal chloroma, normal blood counts and absent coagulopathy. Finally, we present a striking discrepancy between results from examination of bone marrow aspirate and histological analysis. In light of the possible fatal repercussions of delayed APL diagnosis, we want to enlarge the repository of atypical APL manifestations to enable swift APL diagnosis even in more complicated cases.

## Data Availability Statement

The original contributions presented in the study are included in the article/supplementary material. Further inquiries can be directed to the corresponding author.

## Ethics Statement

Written informed consent was obtained from the individual(s) for the publication of any potentially identifiable images or data included in this article.

## Author Contributions

DCH, FL, WH, AR and DH treated the patient. IE, KM and DiH performed all imaging (radiology/nuclear medicine). KU analyzed all histopathological examinations. DCH and DH wrote the manuscript. All the authors revised the manuscript critically, approved the final manuscript, and agreed to be accountable for all aspects of the manuscript.

## Conflict of Interest

The authors declare that the research was conducted in the absence of any commercial or financial relationships that could be construed as a potential conflict of interest.

## Publisher’s Note

All claims expressed in this article are solely those of the authors and do not necessarily represent those of their affiliated organizations, or those of the publisher, the editors and the reviewers. Any product that may be evaluated in this article, or claim that may be made by its manufacturer, is not guaranteed or endorsed by the publisher.
